# Chronic kidney disease among high school students of Kinshasa

**DOI:** 10.1186/1471-2369-13-24

**Published:** 2012-05-04

**Authors:** Justine B Bukabau, Jean-Robert R Makulo, Nestor M Pakasa, Eric P Cohen, François B Lepira, Patrick K Kayembe, Nazaire M Nseka, Ernest K Sumaili

**Affiliations:** 1 Democratic Republic of Congo (DRC), Nephrology Unit, University of Kinshasa, PO Box. 123 KIN XI, Kinshasa, DR, Congo; 2 Democratic Republic of Congo School of Public Health, University of Kinshasa, Kinshasa, DR, Congo; 3 Democratic Republic of Congo (DRC) Department of pathology, University of Kinshasa, Kinshasa, DR, Congo; 4 Nephrology Division, Medical College of Wisconsin, Milwaukee, WI, USA

**Keywords:** Equation of reduced renal function, CKD, High school students, Prevalence, Kinshasa

## Abstract

**Background:**

Chronic kidney disease (CKD) is a major worldwide health problem. However, its burden among adolescents and young adults is unknown, especially in sub-Saharan Africa. The aim of this study was to investigate its prevalence in the school environment. The concordance of usual formulas used to estimate renal function was also assessed.

**Methods:**

In an epidemiological cross sectional study, a random sample of 524 pupils (263 boys, mean age of 18.7 ± 1.4 years) from school environment of Kinshasa were studied. Recorded parameters of interest were anthropometric, proteinuria, serum creatinine and estimated glomerular filtration rate (eGFR) according to the Schwartz formula using uncalibrated creatinine levels from one random measurement. CKD was defined as the presence of kidney damage (daily proteinuria ≥ 300 mg) and/or reduced kidney function (eGFR < 60 ml/min/1.73 m^2^). Concordances between eGFR according to Schwartz, Cockcroft-Gault (C-G) indexed for BSA and modification of diet in renal disease (MDRD) study equations were computed using the kappa coefficient.

**Results:**

The prevalence of CKD by the Schwartz formula was 1.5%. By stage, 0.8% had CKD stage 1 (proteinuria with normal eGFR) and 0.8% had CKD stage 3 (eGFR, 30 to 59 ml/min/1.73 m^2^). The prevalence of proteinuria ≥ 300 mg/day was 1% (one case had 2.7g/day). Agreement between eGFR according to Schwartz formula and the MDRD formula was excellent (kappa: 88.8%). Although correlations between all formulas were excellent (0.99; 0.87, and 0.89), agreement was poor between eGFR according to Schwartz and C-G indexed BSA equation (kappa: 52.7%) and, poorer with C-G unadjusted for BSA (kappa: 26.9%).

**Conclusion:**

In the large African city of Kinshasa, 2% of high school students have CKD. This high prevalence rate emphasizes the need for appropriate detection and prevention measures in this vulnerable young age population group.

## Background

Chronic kidney disease (CKD) is a worldwide public health concern [1]. Epidemiological studies in both western and developing countries have reported an increase of its incidence and prevalence in recent years [1,2]. People with CKD may develop cardiovascular illness more often than unaffected general population, and may also progress to end stage renal disease (ESRD) [3]. At that late stage, management requires dialysis or kidney transplantation which are extremely expensive, over 30 billion dollars yearly in the United States [4]. The cost of ESRD accounts for 0.7% and 1.8% of the English and Belgian health budgets, respectively [5].

Thus, prevention and early detection of CKD have been advised in many guidelines [6]. Early detection can be effective in developed countries, but it is problematic in developing one’s like the Democratic Republic of Congo (DRC) where recent reports have shown that majority of patients are only detected at late stages [7]. Indeed, about 75% CKD patients admitted to the Renal Unit of the University Hospital of Kinshasa are at stages 4 and 5 of kidney disease [8].

In children, CKD is frequently due to congenital malformations of the kidneys and the urinary tract, and inherited disorders [9]. Glomerulonephritis due to infections is a major cause of CKD in Africa [10]. However, paucity of renal symptoms and signs in early stages of CKD hinders their early detection. The tests performed to detect CKD at early stage are urinalysis, proteinuria, calculated creatinine clearance or estimated glomerular filtration rate (eGFR) and imaging of the renal parenchyma and urinary tract [11]. The GFR is a useful tool to assess the progression of CKD. However, the precise measurement of GFR is invasive, time-consuming, expensive and technically difficult to perform, especially in children and adolescents. To bypass these constraints, the Schwartz formula has been recommended to estimate GFR in children and adolescents [12]. In routine practice, pediatricians often do use Cockcroft-Gault (CG) or Modification of Diet in Renal Disease (MDRD) formulas instead of the Schwartz formula, especially for adolescents [13,14].

In Congolese HIV-infected children (median age 7 years), the prevalence of proteinuria is reported to be of 23.8% [15]. However, knowledge of CKD among non-HIV-infected young Congolese is limited. In order to ascertain the burden of CKD in Congolese children, the present study was designed to determine the prevalence of CKD among adolescents and young adults in the school environment of Kinshasa. The agreement between Schwartz, CG and MDRD formulas was also tested.

## Methods

In this cross sectional study, a sample of 524 secondary school students was randomly selected according to a multistage sampling. The units of the survey were: East of the city of Kinshasa (first degree), urban districts (second degree) and schools (third degree). A total of six schools were visited. The proportions of participants varied according to the class of school.

Students were examined by the research team, who recorded demographic parameters (age and sex), body weight, height and waist circumference. The body mass index (BMI) was calculated from the measured weight (in kilograms) and height (in meters) and was categorized as not obese (< 25 kg/m^2^), overweight (25–29, 9 kg/m^2^) or obese (≥ 30 kg/m^2^) according to the WHO 2000 criteria [16]. Blood pressure was measured three times in a sitting position, in the right or left arm, at the heart level, using a calibrated aneroid sphygmomanometer. All students could relax for 5 minutes before determination of blood pressure and an average of three blood pressure measurements was used. Hypertension (HTN) was defined as systolic blood pressure (SBP) ≥ 140 mmHg or diastolic blood pressure (DBP) ≥ 90 mmHg and/or concomitant use of antihypertensive medications by self-report.

We collected a 5 ml blood sample for serum creatinine measurement using the Jaffe compensated method. The GFR was estimated by Schwartz formula and compared to the CG equation normalized per 1.73 m^2^ of BSA and to the MDRD Study formula.

The participants provided a urine sample to detect protein by test strips (Albustix). Female subjects were instructed to void a random urine specimen, remote from menstrual periods. Results were expressed as negative, 1+, 2+ or 3+. Twenty-four quantitative proteinuria was measured by Esbach method if proteinuria by dipstick was positive. All measurements of serum creatinine and daily proteinuria were performed in the laboratory of the Belgian medical center of Kinshasa ‘CMK’.

In that present study, proteinuria was defined as a positive reaction to the Albustix test (≥ 1+ albumin equivalent to ≥ 30 mg/dl) which was confirmed by a quantitative proteinuria ≥ 300mg/day.

The Kidney disease improving global outcome (KDIGO) guidelines [6] for definition and classification of CKD were used. CKD all stages was defined as the presence of quantitative daily proteinuria ≥ 300mg and/or an eGFR < 60 ml/min/1.73 m^2^. In brief, CKD stages are defined as follows: stage 1, proteinuria ≥ 300 mg per day with an eGFR ≥ 90 mL/min/1.73 m^2^; stage 2, proteinuria ≥ 300 mg/day with an eGFR of 60–89 mL/min/1.73 m^2^; stage 3, an eGFR of 30–59 mL/min/1.73 m^2^, stage 4, an eGFR of 15–29 mL/min/1.73 m^2^ and stage 5, an eGFR < 15 mL/min/1.73 m^2^.

### Schwartz equation

[K × height in centimeters]/[serum creatinine level in mg/L]. For this study, K was 0.55 and 0.7 for adolescent girls and adolescent boys respectively [17].

### CG equation

[(140-age in years) × weight in kilograms]/[72× serum creatinine level in mg/dL], with use of the 0.850 multiplier for female gender [13]. Predictions by CG equation were also normalized per 1.73 m^2^ of body surface area (BSA) for comparison to the other equations. BSA (m^2^) was calculated as √ height (cm) × weight (Kg)/3600 [18].

### MDRD study equation

186 × serum creatinine level [mg/dL]^-1,154^ × age [years] ^-0,203^. For women and blacks, the product of this equation is multiplied by correction factors of 0.742 and 1.21 respectively) [14]. The equation was used without creatinine assay calibration. Thus, the MDRD formula predictions could be biased by to differences in the creatinine assay between the MDRD laboratory and our laboratory [19].

### Statistical analysis

All data were processed using a standard statistical package (SPSS version 10.05, 2004). The results are presented as means ± standard deviations, and/or proportions. To compare groups, we used the Chi-square test for qualitative variables and Student *t* test for quantitative variables. The agreement between the eGFR by Schwartz versus CG standardized BSA and MDRD equations were assessed by the kappa coefficient. A *p* value < 0.05 indicated statistical significance.

All participants provided informed written consent. This study was approved by the Ethics committee of the Public Health of the University of Kinshasa and the relevant political, administrative and school authorities.

## Results

### Characteristics of the study population

The general characteristics of the entire group are summarized in Table 1. Girls had higher BMI and waist circumference than boys.

**Table 1:** Characteristics of the population

	**Whole Group N = 524**	**Males N = 263**	**Females N = 261**	**pValue**
Age, years ± SD	18.7 ± 1.4	18.7 ± 1.4	18.6 ± 1.4	ns
Weight, Kg ± SD	55.4 ± 8.2	57.8 ± 7.7	52.9 ± 7.9	ns
Height, cm ± SD	167.1 ± 8.9	172.8 ± 7.3	161.4 ± 6.4	0.047
BMI, Kg/m^2^ ± SD	19.8 ± 0.4	19.3 ± 2.1	20.2 ± 2.7	0.012
BMI 25–29 Kg/m^2^, n (%)	15 (2.9)	3 (1.1)	12 (4.6)	<0.0001
BMI ≥ 30 Kg/m^2^, n (%)	5 (0.9)	0 (0)	5 (1.9)	<0.0001
Waist circumference, cm ± SD	78.5 ± 11,3	73.9 ± 9.8	83.3 ± 10.5	<0.0001
SBP, mmHg ± SD	107.3 ± 11.3	108.1 ± 11.7	106.5 ± 10.8	ns
DBP, mmHg ± SD	70.9 ± 8.1	71.7 ± 8.2	70.1 ± 7.9	0.013
HTN, %	16 (3.1)	10 (3.8)	6 (2.3)	0.45

Values expressed as numbers and proportions (%) in parentheses ± SD, as appropriate.

Abbreviations: *HTN* = hypertension, *SD* = standard deviation, *SBP* = systolic blood pressure, *DBP* = diastolic blood pressure. *BMI* = body mass index; *ns* = no significant.

### Prevalence of CKD

Table 2 shows the prevalence of CKD in secondary school students of Kinshasa. Depending on the method used to estimate the eGFR, 1.5%, 1.7%, 2.9% and 7.6% had CKD according to the Schwartz, MDRD, CG indexed BSA and CG formulas, respectively. No subjects were found at later CKD stages 4 and 5.

**Table 2:** Prevalence of CKD in high school students of Kinshasa

	Schwartz	MDRD	CG indexed BSA	CG
	mL/min/ 1.73 m^2^	mL/min/ 1.73 m^2^	mL/min/ 1.73 m^2^	mL/min
1	4 (0.8)	4(0.8)	2 (0.4)	2 (0.4)
2	0	1(0.2)	3 (0.6)	3 (0.6)
3	4(0.8)	4(0.8)	10 (1.9)	35 (6.7)
4	0	0	0	0
5	0	0	0	0
All stage	8 (1.5)	9 (1.7)	15 (2.9)	40 (7.6)

Values expressed as numbers and proportions (%) in parentheses.

CKD is defined as either kidney damage (proteinuria ≥ 300 mg/day) and/or kidney function (< 60ml/min/1.73 m^2^). KDIGO CKD stages are defined as follows: stage 1, proteinuria ≥ 300 mg per day with an eGFR ≥ 90 mL/min/1.73 m^2^; stage 2, proteinuria ≥ 300 mg/day with an eGFR of 60–89 mL/min/1.73 m^2^; stage 3, an eGFR of 30–59 mL/min/1.73 m^2^, stage 4, an eGFR of 15–29 mL/min/1.73 m^2^ and stage 5, an eGFR < 15 mL/min/1.73 m^2^.

The prevalence of proteinuria by dipstick was 7.4% (39 students). Only five students (1.0%) had proteinuria ≥ 300 mg/day, and the amounts of this proteinuria were 370, 400, 490, 1050 and 2700 mg. By multivariate logistic regression analysis we found no association between proteinuria or reduced kidney function and risk factors such as hypertension, diabetes and obesity.

### Indicators of renal function by Schwartz equation

The indicators of renal function are shown in Table 3. Girls had average serum creatinine levels higher than that recorded in boys.

**Table 3:** Indicators of renal function according to the Schwartz formula in high school students from Kinshasa

	Whole group**N** = 524	**Males N = 263**	**Females N = 261**	**P value**
S. creatinine, mg/dL ± SD	0.92 ± 0.20	0.87 ± 0.16	0.96 ± 0.21	<0.0001
eGFR, mL/min/1.73 m^2^ ± SD	119.13 ± 33.02	143.75 ± 24.87	96.32 ± 21.08	<0.0001
eGFR ≥ 90 mL/min/1.73 m^2^, %	77.9	99.6	57.7	
eGFR 60–89 mL/min/1.73 m^2^, %	21.4	0.4	40.8	
eGFR < 60 mL/min/1.73 m^2^, %	0.8	0	1.5	

Values are proportions or mean ± SD, as appropriate. SD = standard deviation.

### Comparison between Schwartz formula, CG indexed BSA and MDRD study equations

**Figure 1:**
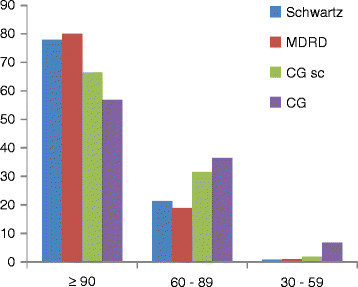
Comparison of eGFR between Schwartz versus MDRD study, CG indexed BSA and CG unadjusted for BSA.

Figure 1 compares the eGFR by Schwartz versus CG indexed BSA and MDRD study. By grouping together the pupils with regard to their eGFR, the results obtained with the Schwartz formula were close to those obtained by MDRD study equation.

The linear correlation was excellent between Schwartz vs MDRD formula (r = 0.99), Schwartz vs CG indexed BSA (r = 0.87) and for Schwartz vs CG formula not indexed to BSA (r = 0.89). However, the concordance between Schwartz, CG indexed BSA and MDRD study formulas to define eGFR < 60 ml/min/1.73 m^2^, according to kappa statistics showed very good agreement only between Schwartz and MDRD (kappa = 0. 88) (Table 4).

**Table 4:** **Correlation of eGFR < 60 ml/min/1.73 m**^**2**^**based on different equations and agreement between formulas**

	Schwartz	
	correlation	kappa
MDRD study equation	0.99	0.888
CG indexed BSA	0.87	0.527
CG formula	0.89	0.269

MDRD: Modification of Diet in Renal Disease CG: Cockcroft - Gault.

## Discussion

Our study documents for the first time the burden of CKD based on KDIGO guidelines in presumed healthy high school students in Kinshasa. We show that CKD affects Congolese people at school age. The overall prevalence of CKD in this population varies between 1.5-2.9% depending on the method used to estimate GFR. The difference observed in the prevalence of CKD according to Schwartz versus CG equations may derive from the body weight measurement, a variable that is not included in the Schwartz formula [12]. Thus, relative lower body weight in school children may explain some of higher prevalence of CKD stage 3 according to the CG formula. Hence, we considered only the results based on Schwartz formula. Based on this method, 1.5% of pupils are affected by all stages of CKD. This finding is eight times less than the 13% prevalence of CKD yielded in American adults [20] and is also much less than the 12.4% previously recorded in adult general population from Kinshasa [2]. However, it is similar to the prevalence of 1.4% in the age group between 20 and 39 years [2], regardless of the time of study and the formula used to estimate GFR (uncalibrated versus calibrated serum creatinine).

Nearly 1% of this pediatric population had CKD stage 3. This result is lower than that of 1.7% recorded among children in Mexico [21]. Comparisons between studies are difficult as the definitions and the criteria of selection may differ. Although the present study did not address the specific etiology of CKD, none of the subjects at CKD stage 3 had hypertension, diabetes or obesity. Possible causes could include genetic or acquired kidney disease.

At a similar age in this survey, girls had an average serum creatinine levels higher than that of boys, which corresponded to an average of lower eGFR. The reason for this discrepancy remains unclear, but might due to differences in sexual maturity and activity. Indeed, Congolese girls reach sexual maturity and activity earlier than Congolese boys [22]. It is likely that some girls in our survey were sexually active, a fact that could account for higher relative muscle mass and/or even HIV infection. Moreover, HIV prevalence is higher among young women than in young men [23,24]. It is also known that young women have a great susceptibility to HIV infection than men [24]. By contrast, the higher BMI observed in females, which is likely mainly driven by fat, but not muscle, should not contribute to higher serum creatinine levels. However, this study did not address the specific causes of lower eGFR in individual subjects.

Another important finding in this present study is the excellent agreement between both Schwartz versus MDRD study equations contrary to CG in the age ranges of 16 to 24 years. To the best our knowledge, these equations have not been validated in African populations. That good concordance found from 16 years old could be explained by the fact that both formulas do not consider the weight. Consequently, in the absence of a reference test, the MDRD or Schwartz formulas are useful tools for the estimation of GFR in adolescents. More structured studies comparing the MDRD study equation to a reference test such as Inulin, Iothalamate or Iohexol could be conducted to validate their correspondence to the real GFR. But, such expensive testing may not be very helpful. Also, the accuracy of GFR estimating equations is known to be highly dependent on creatinine assay, which should be calibrated with that of the MDRD laboratory for use in the MDRD study equation [19] as well as for the more recent new equations for children [25] and the CKD-EPI equation for adults > 18 years [26]. Thus, many centers in industrialized countries move towards enzymatic creatinine, which yield lower creatinine values. This is not the case in many low income countries like the DRC, where many laboratories still use the Jaffe method. Consequently, the new equations are not appropriate for us. Hence, our option to use older equations appears justified and relevant for some low resourced countries.

Obesity and hypertension were infrequent among the students in this study. The prevalence of 0.9% of obesity is similar to the data reported by Kane *et al.* in Senegal [27]. Despite of the ‘epidemiological transition’, the low prevalence of obesity in developing countries can be explained by the fact that adolescents and young adults still face economic burden, political, cultural and religious upheavals’ different from those encountered in developed countries. The prevalence of 3.1% for hypertension is consistent with results from previous results observed in schools of Kinshasa, which ranged between 1.4 to 5% [28].

Our observations show that prevalence of qualitative and quantitative proteinuria was 7.4% versus 1%, respectively. We found one case of quantitative proteinuria of 2.7 grams/day. Some cases with qualitative proteinuria could just be intermittent as during orthostatic proteinuria frequently reported in children and adolescents. Studies in North America, Europe and Asia have shown that prevalence of proteinuria in children varies according to the methodology used. This may have important implications for the cost effectiveness and viability of a screening program [29].

This study had some limitations that must be acknowledged when interpreting the results. Although all positive dipstick proteinuria (qualitative) tests were supplemented by quantitative daily proteinuria as an indicator of kidney damage, only one single measurement was done. Studies based on NHANES III indicate that in repeated measurement, only 63% of those with albuminuria will have persistently positive results [20]. In KDIGO guidelines [6], the definition of CKD requires the persistence of kidney damage for at least 3 months. Hence, the single measurement of proteinuria, serum creatinine or BP in our study, might overestimate the prevalence of CKD and HTN.

On the other hand, the prevalence of CKD may have been underestimated because urinalysis was not done to detect hematuria and renal imaging was not routinely performed.

## Conclusion

Our study establishes that roughly 2% of high-school students in Kinshasa have proteinuria and/or estimated GFR < 60 ml/min. There is a good agreement between eGFR by Schwartz versus MDRD study equation in our target population. This high prevalence appeals for awareness and action like systematic screening for CKD to alleviate the burden of CKD in the schools and the identification of preventable risk factors specific to this age group in order to address and slow down the progression to ESRD.

## Competing interests

The authors declared that they have no competing interest.

## Authors’ contributions

JB designed, acquired data, analyzed, interpreted data, drafted and revised the manuscript. JRM acquired data, analyzed, interpreted data, drafted and revised the manuscript, EKS analyzed, interpreted data, drafted and revised the manuscript. EPC revised the manuscript. FBL interpreted data, drafted the manuscript. PKK designed, analyzed, and interpreted data. NMN designed interpreted data and revised the manuscript. NMP interpreted data, drafted and revised the manuscript. All authors read and approved the final manuscript.
